# Crude Methanol Extract of *Echinophora Platyloba *Induces Apoptosis and Cell Cycle Arrest at S-Phase in Human Breast Cancer Cells

**Published:** 2018

**Authors:** Elnaz Birjandian, Nasrin Motamed, Narguess Yassa

**Affiliations:** a *Department of Cellular and Molecular Biology, School of Biology, College of Science, University of Tehran, Tehran, Iran.*; b *Department of Pharmacognosy, Faculty of Pharmacy, Tehran University of Medical Sciences, Tehran, Iran.*

**Keywords:** *Echinophora platyloba*, MDA-MB-231 cells, Cytotoxic activity, Cell cycle arrest, Apoptosis

## Abstract

The aim of the present study was to determine cytotoxic activity of crude methanolic extract of *Echinophora platyloba* on breast cancer MDA-MB-231 cell line. The free radical scavenging effects of methanolic extract of *E. platyloba *were tested using DPPH method. Crude methanolic extract exhibited potential antioxidant activity with an IC_50_ value of 234.28 ± 21.63 μg/mL when compared to the standard BHT with an IC_50_ value of the 19.5 ± 0.8 μg/mL.

In addition, the *in-vitro* cytotoxic activity of this extract was studied against MDA-MB-231 and MCF-10a cells by MTT assay for 12, 24 and 36 h. Our data showed 534.6 ± 7.2 μg/mL of extract following 24 h of incubation was the most cytotoxic dose against MDA-MB-231 cells in comparison with other doses. This extract could induce apoptosis and promote cell-cycle arrest at S-phase in MDA-MB-231 cells after 24 h of incubation, as compared to the control group (*p *< 0.001) and could significantly up-regulate the expression of bax and p27 genes at the level of 2.8 and 2.2 folds, respectively. While, a significant amount of down-regulation was observed for bcl-2 gene expression, which was observed to be 0.4 fold. The present results prove the anticancer capacity of crude methanolic extract of *E. platyloba *to inhibit limit cell proliferation, and inducing cell cycle arrest and apoptosis.

## Introduction

Breast cancer is a serious global health concern, being the second most common of all cancers and by far the most frequent cancer amongst women (1). The usual treatment strategies for breast cancer are surgery, radiotherapy, chemotherapy, immunotherapy and the use of traditional Chinese medicine. However, these therapies exert a serious amount of side effects (2-3). Thus, it is important to find a new high efficacy and low toxicity alternative for the treatment of this disease. 

For many years, the anti-proliferative actions of chemotherapeutic drugs were ascribed solely to their ability to induce genotoxic damage. Natural plant components are excellent sources of complex chemicals with useful properties, including great therapeutic value (4). To date, many anticancer drugs have been developed from different sources and being prescribed by clinicians (5). 

The genus Echinophora belongs to Umbelliferae family and is represented in the Flora of Iran by four species *E. cinerea*, *E. platyloba*, *E. orientalis* and *E. sibthorpiana*. Two former species are exclusive of Iran (6). *E. platyloba *is widely used as a food seasoning and edible vegetable in western and central Iran where this plant is known as ‘‘Khousharizeh’’ or “Tigh Touragh” (7). The plant-derived food spices not only improve the flavor of foods but also have positive effects on our health. They may eliminate pathogenic microorganisms, act as a chemo-preventive agent against different cancers and may exhibit other biological activities and thus are being used for the treatment of a number of human ailments (7-8).

Previous studies have reported that *E. platyloba* extract has antifungal, antimicrobial and anticancer activities (9-13). Keeping in mind the significance of herbal extracts, the present study aims to investigate some biological effects of the crude methanolic extract of *E. platyloba* leaves. The anticancer, apoptosis inducing and cell-cycle altering effects of the present extract were observed against MDA-MB 231 breast cancer cell line.

## Experimental

*Plant material collection*

The fresh plant material was collected in May 2014 from the region of around Golpayegan, Isfahan province, Iran. The present sample was identified and authenticated by the taxonomist and a voucher authentication number of 45816-Tuh was assigned and deposited in the Department of Biological Sciences, University of Tehran, Tehran, Iran.

*Extraction of plant material*

Collected plant material was dried at room temperature; its leaves were removed from the stem and were then powdered. Powdered leaf samples were extracted by floating them in methanol. The crude methanol extract of *E. platyloba* leaves was prepared by weighing 300 g of pre-prepared powder and extracting the power with maceration method by using 96% methanol eluent. Extraction was conducted under ambient temperature and the eluent was replaced every 24 h. The extraction process (eluent replacement) was repeated 10 times and the resultant extract was filtered by Watman filter paper. The macerate was evaporated by vacuum rotary evaporator under a temperature of 50 °C to obtain thick greenish matrix. The crude methanolic extract was then stored for the further experiments. 

*DPPH radical scavenging activity method*

The free radical scavenging activity of crude methanol extract was evaluated using the stable radical DPPH. A series of extracts with different concentrations (62.5-1000 µg/mL) were prepared for this purpose. Then, 2.5 mL of each concentration and 1.0 mL of DPPH solution in methanol were mixed and placed in the dark at the room temperature for 30 min. The absorbance of each extracted sample was observed after the addition of DPPH into it and its absorbance was measured at 517 nm using UV–Visible Lambda spectrophotometer. Methanol (1.0 mL) plus the plant extract solution (2.5 mL) were used as the blank, while the DPPH solution plus methanol were used as the control. Butylated hydroxytoluene (BHT) was used as a positive control in this study. All the experiments were performed in triplicate. The DPPH free-radical scavenging activity, in terms of DPPH (%), of crude methanol extract was determined using the following relation: 

DPPH free-radical scavenging capacity (%) = (1 – (Ab_of sample_ – Ab_of blank_)/Ab_of control_) × 100.

The antioxidant activity of crude methanolic extract was partially expressed as IC_50_, which was defined as the concentration of extract required to inhibit the formation of DPPH radicals by 50%. 

*Cell culture and treatment*

MDA-MB-231 and MCF-10a cells were cultured in cell culture flask at 37 °C with 5% CO_2_ in RPMI 1640 medium with 10% fetal bovine serum (FBS) and 1% antibiotics (penicillin-streptomycin). 

Different concentrations (25-1000 g/mL) of crude methanolic extract were prepared by dissolving it into DMSO and then diluting it with RMPI medium under sterile conditions.

Cultured cancer cells with 80-90% confluence were used for plating. The adherent cells were detached from the flask bottom by trypsinization and were seeded at the quantity of 100 µL of cells per well of the 96-well microtiter plates (1 - 5 × 10^4^ cells/well). The plates were maintained at 37 °C in a humid incubator with an air mixture containing 5% (v/v) CO_2_ for 24-48 h until 80-90% confluence. Then, old medium were discarded and 200 µL of new medium were added. Then, the cells were treated with serial dilution of crude methanolic extract and toxol. Toxol was used as standard drug. The cells were also treated with DMSO as negative control. The plates were returned to incubator for 12, 24 and 36 h. All treatments were done in triplicate. 

*MTT assay of in-vitro cytotoxicity*

After treatment with crude methanol extract, the cell viability was evaluated using MTT assay. Cell samples were incubated with 25 μL MTT for 3 h at 37 °C, the supernatant was removed and the formazan crystals, formed in viable cells, were solubilized with 100 μL of DMSO. Finally, the absorbance of each well was measured at 570 nm with ELISA Reader. The percentage growth inhibition was calculated using following relation: 

% cell inhibition = 100 - ((At - Ab)/(Ac - Ab)) × 100

where, At = absorbance value of test compound, Ab = Absorbance value of blank and Ac = Absorbance value of control.

The anticancer effect and effective dosage of present extract was evaluated in terms of IC_50_ values (the drug concentration affecting the growth of treated cells by 50% with respect to untreated cells).

**Table 1:** Comparison of cytotoxicity activity of (A) *E. platyloba* and (B) Taxol in 24 h.

Concentration (μg/mL)	Growth inhibition (%)
***(A*** *) E. platyloba*	
**100**	25.35
**200**	32.44
**300**	36.94
**400**	41.97
**500**	48.07
**600**	50.89
**700**	59.63
**800**	60.12
**900**	73.67
**1000**	79.98
*(B) Taxol*	
**5**	47.25
**10**	64.36
**15**	71.15
**20**	79.83
**25**	83.28

**Figure 1:**
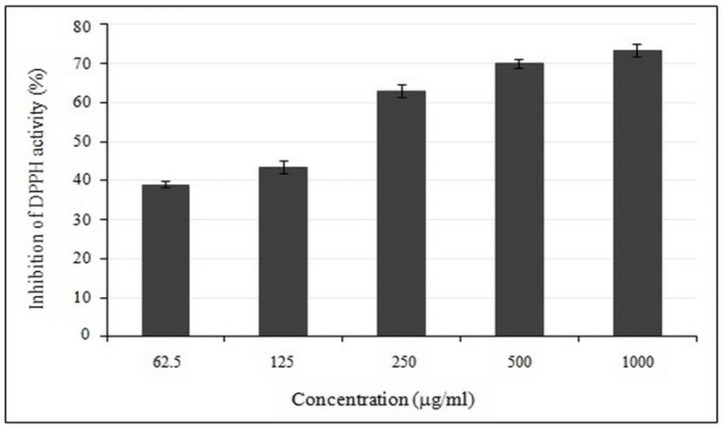
Dose-dependent activity of crude methanolic extract of *E. platyloba* using DPPH radical scavenging assay. Data are mean ± SD (n = 3

**Figure 2:**
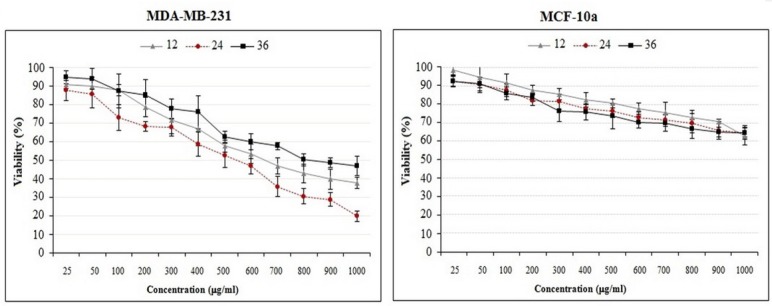
The effects of crude methanolic extract of *E. platyloba* on the proliferation of MDA-MB-231 and MCF-10a cells. Cells were incubated with increasing concentrations of present extract for 12, 24 and 36 h. The proliferative response was then assessed by MTT assay. The data are presented as the means ± SD (n = 3

**Figure 3:**
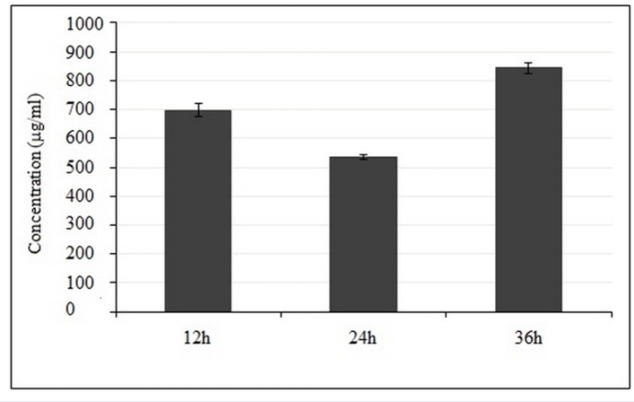
**Activity against the breast cancer cell line MDA-MB-231 with crude methanolic extract of** ***E. platyloba*** **following 12, 24 and 36 h incubations, as assessed using the MTT assay. Data are reported as IC**_50_ **versus time lapse. Graph represents means ± SD from 3 independent experiments**

**Figure 4:**
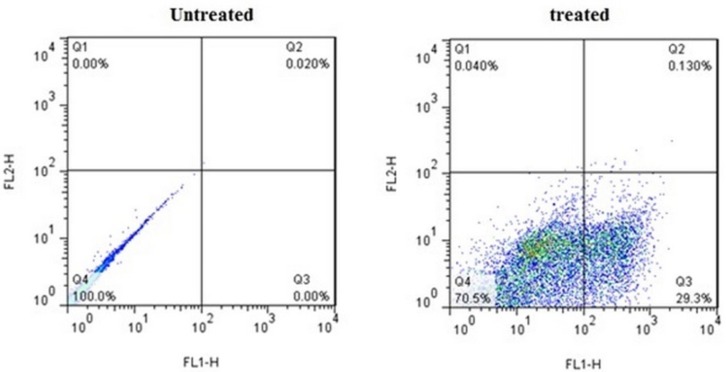
Flow cytometric analysis of Annexin V-FITC (FL1-H) at X-axis and PI (FL2-H) at Y-axis after double staining of MDA-MB-231 cells, treated with crude methanolic extract of *E. platyloba* at 24 h. Alive cell (Annexin V−/PI−) populations were located in the lower left quadrant (LL), apoptotic cell (Annexin V+/PI−) populations in lower right quadrant (LR), late apoptotic (Annexin V+/PI+) populations in upper right quadrant (UR), and necrotic cells (Annexin V−/PI+) were present in the upper left quadrant (UL). Dot plots of Annexin V/PI staining are shown in A) untreated MDA-MB-231 cells, B) MDA-MB-231 cells treated with IC_50_ concentration of crude methanolic extract

**Figure 5:**
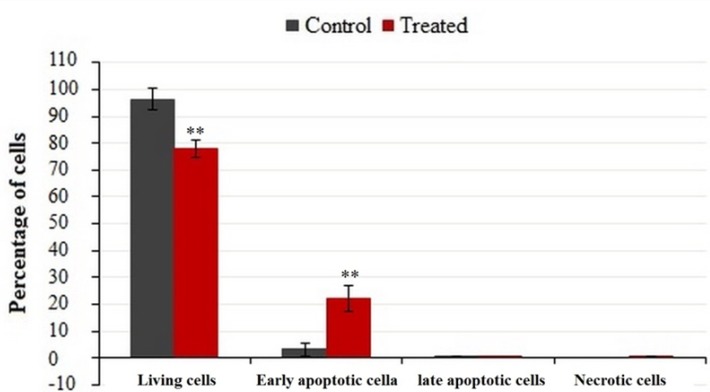
Flow cytometric analysis of phosphatidylserine-annexin V, labeled on the MDA-MB-231 cells, treated for 24 h with IC_50_ of crude methanolic extract. Data represent percentage mean ± SD, n = 3. Results were statistically analyzed with a Student’s *t*-test (*P < 0.05; **P < 0.01; ***P < 0.001

**Figure 6:**
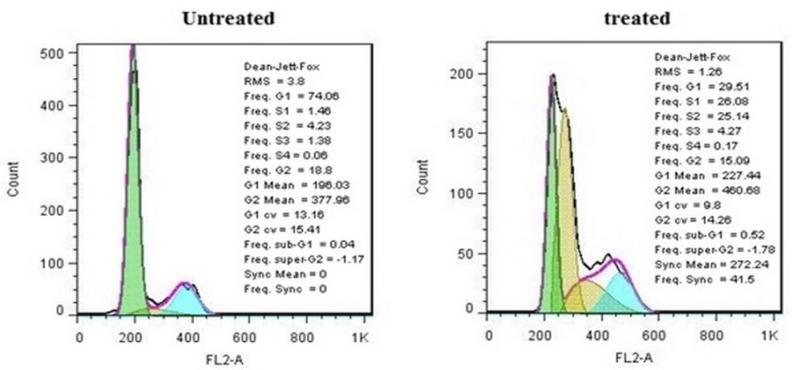
Cell cycle distributions of treated and untreated MDA-MB-231cells

**Figure 7:**
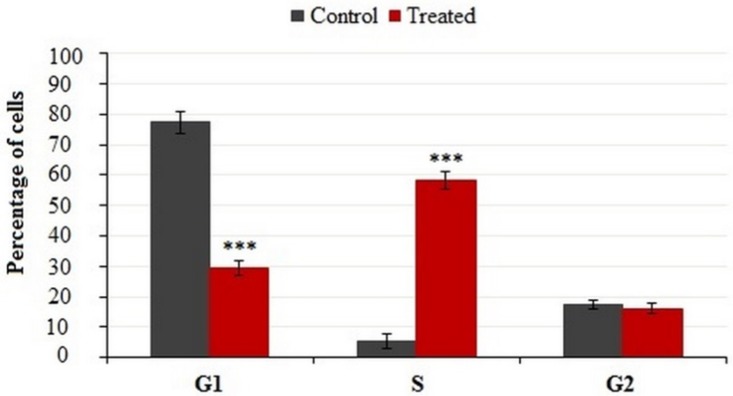
Cells were incubated with IC_50_ concentration of crude methanolic extract for 24 h, harvested and stained with PI and analyzed by flow cytometry. Histograms represented cell cycle distribution. Results were statistically analyzed by student’s *t*-test (*P < 0.05; **P < 0.01; ***P < 0.001

**Figure 8:**
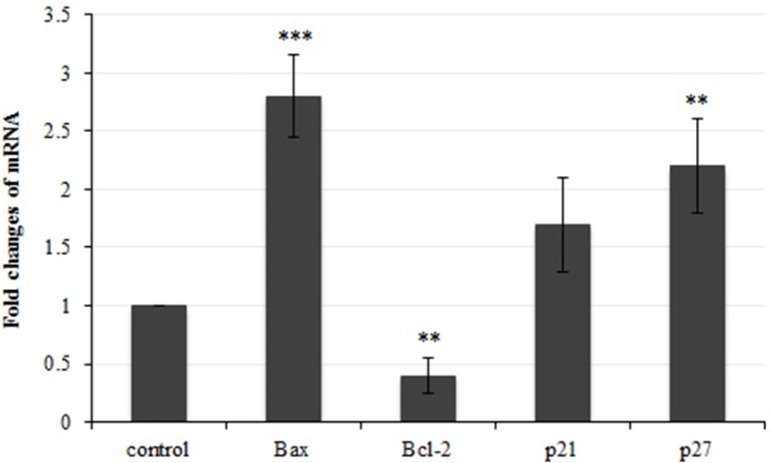
A demonstration of gene expression changes in bax, bcl-2, p21 and p27. Results were statistically analyzed with student’s *t*-test (*P < 0.05; **P < 0.01; ***P < 0.001

*Flow cytometric analysis of apoptosis*

MDA-MB-231 cells were plated in 6-well plates under standard culture conditions. After 24 h, the cells were fed with fresh medium and treated with DMSO alone (control experiment) or administered with IC_50_ concentration of crude methanolic extract. After 24 h of treatment, the culture medium was aspirated; the cells were quickly washed twice with ice-cold PBS and then trypsinized. The resulting cell-pellets were collected for further analyses. For apoptotic determinations, 5 × 10^5^ cells were washed with 1 mL PBS (pH 7.4) and then re-suspended in binding buffer, according to the manufacturer’s protocol. The cell aliquots were then incubated with Annexin-V-FITC and PI solutions and incubated for 15 min at 4 °C in the dark. Then, the apoptotic induction was determined by FACS can flow cytometer with the FlowJo 7.6.1 software. All the experiments were performed in triplicate.

*Cell cycle analysis*

The effect of crude methanolic extract of *E. platyloba* on the survival cells and cell cycle phase distribution of MDA-MB-231 cells was assessed using flow cytometry. Briefly, after treatment of cells with IC_50_ concentration of crude methanolic extract for 24 h, the floating cells were discarded by aspiration and the adherent cells were trypsinized and thereafter washed twice with cold PBS, and centrifuged. The pellet was re-suspended in 50 mL cold PBS and 450 mL of cold methanol for 1 h at 4 °C. The cells were centrifuged at 110×g for 5 min, pellets were washed twice with cold PBS, re-suspended in 500 mL PBS and incubated with 5 mL RNAase at 37 °C for 30 min. The cells were then chilled over ice for 10 min and stained with PI for 1 h for flow cytometric analysis. Flow cytometry was performed with a FACScan flow cytometer with the FlowJo 7.6.1 software*.*

*RNA extraction and RT-PCR analysis*

MDA-MB-231 cells were treated with the IC_50_ concentration of crude methanolic extract for 24 h. Total RNA was extracted from the cell cultures using RNX-Plus Solution, at the 70% cell confluence. A 1 mL of RNX-Plus solution was added to the cell pellet. The reaction mix was then incubated at room temperature for 5 min, followed by the addition of 200 µL chloroform and mixed well. Then, the prepared mixture was incubated at room temperature for 5 min, centrifuged at 12.000×g for 15 min at 4 °C. The aqueous phase was collected into a separate 1.5 mL micro-centrifuge tube and 500 µL of isopropyl alcohol was added to it while mixing. The reaction mixture was incubated again at room temperature for further 10 min and centrifuged at 12.000×g for 10 min at 4 °C. The RNA pellets were acquired by adding 75% absolute alcohol and stored in RNase-free water at -80 °C for further use. The total RNA concentration and its purity were measured using UV-Visible spectrophotometer at the wavelength of 260-280 nm. The integrity of the isolated RNA was confirmed by running electrophoresis of individual samples on 1% agarose gel. 

After RNA preparation, the complementary DNA (cDNA) was reverse-transcribed using the 2-steps RT-PCR kit, according to the manufacturer’s instructions. The synthesized cDNA was immediately used in a real-time PCR or stored at –80 °C for further use.

*Quantitative real time PCR (qPCR)*

The expression of bax, bcl-2, p21, p27 and GAPDH mRNAs was determined using real-time PCR. Each cDNA sample was amplified using SYBR Green on the ABI 7500 Fast Real-time PCR System (Applied Biosystem, CA). The reaction conditions consisted of 2 μL of cDNA + 0.5 μL primers in a final volume of 20 μL of super-mix. PCR reaction parameters were as follows: denaturation at 95 °C for 5 min, followed by 50 cycles of denaturation at 95 °C for 10 sec, annealing at 60 °C for 30 sec, and finally the extension at 72 °C for 30 sec. The whole experiment was performed in triplicate. 

For each sample, the ∆Ct values were determined by subtracting the average of duplicate Ct values of the target gene from the average of duplicate Ct values of the reference gene. The relative gene expression level was also normalized relative to a positive calibrator, consisting of one of the samples from the calibration curve. The relative gene expression level of the calibrator (∆Ct calibrator) was also determined by subtracting the average of duplicate Ct values of the target gene from the average of duplicate Ct values of the reference gene. The results of the present experiment were expressed as ‘N-target’ and determined as follows:

N-target = 2^(∆Ct sample – ∆Ct calibrator)^

*Statistical analysis*

All the data represented in this study are means ± standard deviation (SD) of three identical experiments, carried out in triplicate. Statistical significance of the obtained data was determined by independent *t*-test and the *p*-value ≤ 0.05 was considered significant. 

## Results

*Determination of antioxidant activity*

Use of 2,2-Diphenyl-1-picrylhydrazyl (DPPH) radical scavenging method has been widely used to test the free radical scavenging ability of various dietary samples. The presence of antioxidants neutralizes the DPPH by the transfer of an electron or hydrogen atom. The reduction capacity of DPPH could be determined by color changes from purple to yellow at the wavelength of 517 nm. The crude methanolic extract of *E. platyloba, *tested spectrophotometrically against DPPH reveals that the radical scavenging activity of crude methanolic extract possessed excellent antioxidant capacity by increased with the increasing concentration of the extract (Figure 1). 

The IC_50_ values of crude methanolic extract were found to possess potent free radical scavenging activity (IC_50 _= 234.28 ± 21.63 µg/mL) higher than BHT, a synthetic commercial antioxidant (IC_50 _= 19.5 ± 0.8 μg/mL).

*In-vitro cytotoxic activity of crude methanolic extract of E. platyloba*

The effect of crude methanolic extract was performed on MDA-MB-231 and MCF-10a cells by MTT assay. The normal MCF-10A cells were used as control. The susceptibility of cells to the drug exposure was characterized by IC_50_ values. Results indicate that the anti-proliferative effect of the present extract was strengthened with increase in the concentration of extract. Figures 2 and 3 reveal that crude methanolic extract demonstrates the highest activity against MDA-MB-231 cells with an IC_50_ value of 534.6 ± 7.2 μg/mL during 24 h. Nonetheless, the IC_50_ of crude methanolic extract was higher on the positive control cell line (IC_50_ > 900 μg/mL), MCF-10A as compared to the MDA-MB-231 cells. Cytotoxic effect of the taxol (as standard drug) and the crude methanolic extract of *E. platyloba* on MDA-MB-231 cells after 24 h post-treatment has been presented in 

Table 1.

*Apoptotic induction analysis*

FACS analysis was used to quantify apoptotic or necrotic death after treatment with IC_50_ values of crude methanolic extract from* E. platyloba* for 24 h (Figure 4). Results have demonstrated a statistically significant induction of 23.38 ± 5.12% early apoptosis in MDA-MB-231 cancer cells after treatment with crude methanol extract (Figure 5).

*Cell cycle arrest analysis*

It is well-known that the cell growth inhibition is often accompanied by cell cycle arrest. Many anti-cancer agents arrest the cell cycle at one specific phase and then induce apoptosis. Figure 6 illustrates the effect of crude methanolic extract of *E. platyloba* on the MDA-MB-231 cell line. After 24 h of treatment, the percentage of cells in the S phase significantly increased from 4.58 ± 2.44% to 58.88 ± 3.11% as compared to the control (Figure 7).

*Quantification of mRNA levels of apoptosis-related genes*

To investigate the molecular mechanism of apoptotic induction in the presence of crude methanolic extract of *E. platyloba* in MDA-MB-231 cells, the expression levels of pro- and anti-apoptotic genes were examined. Bcl-2 and Bax are two major proteins, generally involved in apoptosis. It is unknown that if *E. platyloba* extract induces or inhibits the expression of these genes. It is shown in Figure 8 that there was a significant increase in bax expression at 2.8 fold. While, the expression of bcl-2 gene was decreased 0.4 folds when MDA- MB 231 cells were treated with crude methanolic extract after 24 h of incubation as compared to the control cells. Thus, the results indicate that the present plant extract could cause a significant amount of apoptosis in MDA- MB 231 cells mainly via altering the bax and bcl-2 expression.

We next examined the effects of *E. platyloba* on cell cycle regulatory genes in MDA-MB-231 cells (Figure 8). Since inhibitory mechanism of crude methanolic extract on cell proliferation might also affect the expression of negative regulators of the cell cycle, we evaluated gene expression of CDK (cyclin dependent kinase) inhibitor: p21 and p27, which are mainly involved in G1/S transition. Crude methanolic extract of *E. platyloba* induced a significant amount of expression (2.2 fold) in p27 (but not p21) during 24 h of treatment.

## Discussion

In this study, anti-proliferative activity of crude methanolic extract of *E. platyloba* was tested by monitoring cell viability among treated and untreated cells by using MTT assay. Profiles obtained from cell viability in MTT test have showed that the crude methanolic extract of *E. platyloba,* in general, may decrease the viability of MDA-MB-231 cells as compared to the control group. The results have shown that the cytotoxic activity of present extract on MDA- MB-231 cells was more severe than that observed in the MCF-10a normal cells.

Cell cycle progression is an important biological event, having controlled regulation in normal cells, which almost universally becomes aberrant or deregulated in transformed and neoplastic cells (14). In this regards, the potential prognostic role of cell cycle regulators and natural agents’ effects in cancer therapy has been under focus. So, in the present study, the influence of crude methanolic extract of *E. platyloba* was evaluated on the cell cycle mechanism of MDA-MB-231 cells. Data obtained from the cell cycle distribution in the cells, treated with crude extract, were associated with an increase in the cell cycle arrest at S-phase that is known to be controlled by CDK, CDKI, and cyclins (15). Gene expression analysis in this study showed that the crude extract –could induce S-phase cell cycle arrest, which was thought to be mediated through the increased expression of CDKI mRNAs (p27 but not p21). Uncontrolled cell division depends on the activation of cyclins, which bind to CDK to induce cell cycle progression towards the S-phase. CDK activity is one of the major causes of cancer progression and their functions are tightly regulated by CDKI, such as, p21 and p27 proteins. P21 is a universal inhibitor of CDK(s) and p27 is commonly upregulated in response to antiproliferative signals. The increased expression of S-phase cyclins in cancer cells provides an uncontrolled growth advantage because most of these cells either lack CDKI, harbor nonfunctional CDKI, or CDKI expression is not at a sufficient level to control CDK-cyclin activity (16-17).

To further elucidate the pathways of the cell death, induced by the administration of presently prepared crude extract, phosphatidylserine flipping was evaluated using Annexin/PI flow cytometric assay. Exposure of phosphatidylserine on the external surface of the cell membrane is generally accepted as one of the apoptotic biomarkers (18). The Annexin/PI assay confirmed the ability of the crude methanolic extract to induce early and late apoptosis during the present study. Unlike necrosis, apoptosis is an important cell death mechanism that does not trigger an inflammatory response that occasions collateral destruction of normal cells in the surrounding microenvironment (19-20). Thus, apoptosis is a protective mechanism that maintains tissue homeostasis by removing ailing cells (20-21). Cancer cells, however, exhibit resistance to apoptosis in order to sustain their uncontrolled proliferation (22-23) and therefore, any apoptosis modulating compound is desirable as a plausible chemotherapeutic agent against cancer (24). To determine the mechanism of *E. platyloba* extract in inducing apoptotic cell death in MDA-MB-231 cells, we examined the expression of apoptosis-related genes by real time PCR. It has been shown that the bcl-2 family proteins play an important regulatory role in apoptosis, either as activator (bax) or as inhibitor (bcl-2) (25). Of the bcl-2 family members, (26) bax*/*bcl-2 expression level is critical for cell survival or death (27-28). In this investigation, it is found that bax expression was significantly elevated in *E. platyloba* treated MDA-MB-231 cells. While, the bcl-2 mRNA level showed a significant decrease in this experiment, which ultimately results in an increase in the bax/bcl-2 ratio and activation of the caspase cascade. 

## Conclusions

This study addressed that crude methanolic extract of *E. platyloba* inhibited proliferation, induced the apoptotic mechanism and caused cell cycle arrest at S phase in human breast cancer MDA-MB-231 Cells. The molecular events have identified that the efficacy of the presently prepared plant extract was associated with the up-regulation of bax and p21*,* and down-regulation of bcl-2 genes.
